# From Biological Activity to Precision Cannabinoid Oncology

**DOI:** 10.3390/cimb48101019

**Published:** 2026-10-02

**Authors:** Won Sup Lee, Se-Il Go

**Affiliations:** 1 Department of Internal Medicine, Institute of Medical Science, Gyeongsang National University Hospital, Gyeongsang National University College of Medicine, 15 Jinju-daero 816 Beon-gil, Jinju 52727, Republic of Korea; 2 Department of Internal Medicine, Institute of Medical Science, Gyeongsang National University Changwon Hospital, Gyeongsang National University College of Medicine, 11 Samjeongja-ro, Changwon 51472, Republic of Korea; gose1@gnu.ac.kr

**Keywords:** cannabinoids, precision oncology, biomarkers, multi-target activity, cancer therapy, clinical translation

## Abstract

Cannabinoids have gained increasing attention as potential anticancer agents because of their ability to modulate diverse cancer-associated signaling pathways and biological processes.: Cannabinoids, including cannabidiol (CBD) and Δ9-tetrahydrocannabinol (THC), can influence tumor proliferation, apoptosis, angiogenesis, metabolism, immune regulation, and tumor–microenvironment interactions. However, clinical translation remains limited, as evidence of anticancer efficacy is derived predominantly from preclinical studies. Major knowledge gaps include the absence of validated predictive biomarkers, unclear criteria for patient selection, and limited evidence regarding optimal therapeutic and combination strategies. Thus, the central challenge is to identify the patients and tumor contexts most likely to benefit from cannabinoid-based therapy. We propose a precision cannabinoid oncology framework that integrates molecular characterization, molecular classification, predictive biomarker development, rational combination strategies, and prospective clinical validation. This approach may provide a conceptual foundation for moving cannabinoid oncology from broadly empirical investigation toward molecularly informed patient selection and prospective validation of cannabinoid-based therapies in molecularly defined patient populations.

## 1. Introduction

Cannabinoids have attracted considerable interest as potential anticancer agents because they can modulate multiple biological processes involved in tumor growth, survival, metabolism, stress responses, angiogenesis, and the tumor microenvironment [1,2,3]. Preclinical studies across diverse malignancies have reported effects on proliferation, apoptosis, autophagy, oxidative stress, and tumor progression, supporting continued investigation of the endocannabinoid system as a therapeutic target [1,2,3].

However, the biological effects of cannabinoids are highly context-dependent. Responses vary according to cancer type, cannabinoid compound, concentration, receptor expression, downstream signaling, and cellular state [1,2,3]. Consequently, the extensive preclinical evidence of anticancer activity does not necessarily imply uniform therapeutic efficacy across malignancies. This discrepancy highlights a central challenge for clinical development: which tumors are intrinsically vulnerable to cannabinoid-based interventions, and which molecular features determine sensitivity or resistance?

Conventional oncology classification based primarily on histology may therefore be insufficient for cannabinoid therapy. Similar to the development of biomarker-driven targeted therapies, molecular characterization may provide a framework for identifying tumor-specific biological states associated with therapeutic response.

Importantly, “cannabinoids” should not be considered a pharmacologically homogeneous therapeutic class. Phytocannabinoids such as Δ9-tetrahydrocannabinol (THC) and cannabidiol (CBD), synthetic cannabinoid receptor agonists or analogs, and agents that modulate endocannabinoid metabolism, such as fatty acid amide hydrolase (FAAH) or monoacylglycerol lipase (MAGL) inhibitors, differ in receptor activity, target engagement, pharmacokinetics, and downstream biological effects [1,2,3,4,5]. Accordingly, molecular determinants of response identified for one cannabinoid intervention should not automatically be extrapolated to other agents or pharmacological classes.

Therefore, precision cannabinoid oncology should link tumor-specific molecular characteristics not simply to “cannabinoid sensitivity” in general, but to sensitivity to a defined cannabinoid intervention with a specified pharmacological mechanism, formulation, and exposure profile.

We propose that the next phase of cannabinoid oncology should move from empirical administration toward molecularly informed therapeutic selection, in which genomic, transcriptomic, proteomic, and metabolic characteristics are integrated with functional response data to identify tumors most likely to benefit. Here, we define “precision cannabinoid oncology” as a biomarker-informed framework that links tumor-specific molecular characteristics to functional sensitivity to a defined cannabinoid intervention and, ultimately, to therapeutic selection. Depending on the pharmacological class and mechanism of the intervention, candidate determinants may include genomic alterations and expression or activity of cannabinoid receptors and endocannabinoid system components, molecular dependencies in cannabinoid-modulated oncogenic pathways, metabolic and cellular stress states, and compensatory signaling programs associated with resistance [2,3,5,6]. These molecular features should therefore be interpreted in conjunction with functional response data rather than as isolated predictors [7,8,9]. Accordingly, the proposed framework progresses from molecular characterization and functional response assessment to response-associated molecular classification, candidate biomarker development, therapeutic selection, and prospective clinical validation.

Despite extensive preclinical investigation, the strength and reproducibility of the evidence supporting anticancer activity remain heterogeneous. Many reported effects derive from in vitro studies using isolated cancer cell lines and experimental cannabinoid concentrations that may not directly reflect clinically achievable exposure. Findings may also vary according to cannabinoid compound, formulation, dose, exposure duration, receptor context, and tumor model. Evidence from patient-derived models and clinical studies remains comparatively limited, and the available clinical evidence does not currently establish cannabinoids as routine cancer-directed therapy [7,8,10]. These limitations highlight the need to distinguish biological plausibility from reproducible therapeutic vulnerability and clinically validated predictive biomarkers.

## 2. Multi-Target Activity Creates Biomarker Challenges

Cannabinoids can influence multiple cancer-associated processes, including proliferative signaling, cell death, metabolism, angiogenesis, invasion, and immune–microenvironmental interactions [1,2,3]. Representative biological effects and associated signaling pathways are summarized in Table 1. The broad biological activity of cannabinoids represents both an opportunity and a challenge for precision oncology. Unlike therapies directed against a defined oncogenic alteration, cannabinoids can influence multiple receptors, signaling pathways, metabolic processes, and cellular stress responses simultaneously [1,2,3]. A single universal biomarker is therefore unlikely to explain the full spectrum of cannabinoid responsiveness.

Several tumor-intrinsic features may contribute to this variability. CB1 (CNR1) and CB2 (CNR2) can influence proliferation, survival, apoptosis, inflammation, and tumor–immune interactions, although their biological effects vary substantially across tumor contexts [2,3]. Similarly, endocannabinoid-metabolizing enzymes such as FAAH and MAGL regulate the availability of endogenous ligands and lipid-derived signaling molecules and may contribute to differences in tumor sensitivity [3,4,5].

Cannabinoid signaling also intersects with oncogenic pathways including PI3K/AKT/mTOR, MAPK, EGFR, and Wnt/β-catenin, potentially modifying cellular survival and stress responses [2,3]. However, pathway activation alone should not be interpreted as evidence of cannabinoid sensitivity. Rather, the degree of pathway dependency and the capacity of tumor cells to activate compensatory mechanisms may determine whether pathway perturbation translates into therapeutic vulnerability.

Tumor context adds another layer of complexity. Immune-cell composition, inflammatory signaling, stromal interactions, hypoxia, and metabolic state may modify cannabinoid effects [3,11]. Furthermore, intratumoral molecular heterogeneity and clonal evolution can generate distinct populations with different pathway dependencies and treatment responses [12].

Thus, the central biomarker challenge is not simply identifying molecular features associated with cannabinoid activity, but determining which combinations of molecular features define a therapeutically vulnerable tumor state.

## 3. Heterogeneity of Cannabinoid Response and Molecular Determinants

Substantial heterogeneity in cannabinoid response has been observed across tumor types and experimental models [1,2,3]. Differences in receptor expression, downstream signaling, metabolic state, and cellular stress responses may contribute to variable sensitivity, including heterogeneity within the same histological cancer type [1,2,3]. Most evidence supporting these mechanisms derives from in vitro and preclinical models [2,3]. However, the extent to which these findings translate into clinically meaningful anticancer activity remains uncertain, and current clinical evidence is insufficient to establish cannabinoids as routine cancer-directed therapy [10]. Thus, histological classification alone may be insufficient to predict cannabinoid responsiveness, highlighting the need to identify molecular determinants that distinguish sensitive from resistant tumors. Representative cancer-specific differences in cannabinoid interventions, proposed mechanisms, candidate biomarkers, and evidence maturity are summarized in Table 2.

### 3.1. Genomic Determinants of Cannabinoid Sensitivity

#### 3.1.1. Which Genomic Alterations Confer Susceptibility or Resistance to Cannabinoid-Based Therapies?

A central question in precision cannabinoid oncology is whether specific genomic alterations confer susceptibility or resistance to cannabinoid-based therapies. Although cannabinoids can modulate multiple cancer-associated signaling networks, evidence directly linking individual genomic alterations to reproducible cannabinoid sensitivity remains limited [2,3]. Potential determinants may include alterations affecting the endocannabinoid system, oncogenic signaling networks, cell-death regulation, and metabolic pathways.

Genomic alterations affecting CNR1 and CNR2 have been identified across multiple cancer types, and recent genomic analyses have described recurrent or truncating variants that may affect receptor structure or intracellular signaling; however, their relationship with cannabinoid sensitivity remains unvalidated [6]. Alterations affecting FAAH and MGLL may also modify endocannabinoid metabolism and cannabinoid-related signaling, providing a biological rationale for investigating these genes as candidate determinants of therapeutic response [3,4,5]. However, these alterations have not been established as validated predictive biomarkers for cannabinoid-based anticancer therapy.

Similarly, genomic alterations affecting oncogenic pathways such as EGFR, PI3K/AKT/mTOR, RAS/RAF/MAPK, and Wnt/β-catenin may modify the cellular consequences of cannabinoid-mediated signaling perturbation [2,3]. However, the presence of an alteration within these pathways should not be interpreted as evidence of cannabinoid sensitivity, because therapeutic vulnerability is likely to depend on the degree of pathway dependency, cellular context, and activation of compensatory survival mechanisms.

Thus, an important distinction should be made between genomic alterations associated with cannabinoid-related biology and genomic alterations that predict therapeutic benefit. The current evidence is largely based on mechanistic and preclinical studies, and reproducibility across tumor types, experimental systems, and defined cannabinoid interventions remains insufficiently established. At present, no broadly validated genomic alteration can be considered a predictive biomarker for anticancer cannabinoid therapy. Identifying such determinants will require integration of genomic profiling with functional cannabinoid-response assays and subsequent clinical validation.

#### 3.1.2. Can Genomic Profiles Identify Tumor Subsets with Intrinsic Vulnerability to Cannabinoid-Mediated Signaling Modulation?

Beyond individual genomic alterations, an important question is whether integrated genomic profiles can identify tumor subsets with intrinsic vulnerability to cannabinoid-mediated signaling modulation. Because cannabinoid activity involves multiple receptors, signaling pathways, metabolic processes, and cellular stress responses [2,3], a single genomic alteration may be insufficient to predict therapeutic response. Instead, combinations of genomic alterations may define molecular states characterized by specific oncogenic dependencies, altered endocannabinoid-system activity, or impaired stress adaptation. Such profiles could provide a basis for identifying candidate cannabinoid-sensitive tumor subsets, but this remains a hypothesis supported primarily by biological plausibility and preclinical evidence rather than by reproducible clinical biomarker studies. Validation will require independent functional testing across multiple models and prospective clinical evaluation.

### 3.2. Endocannabinoid-System Determinants

Alterations in *CNR1, CNR2, FAAH, and MGLL* may influence the functional state of the endocannabinoid system and consequently modify responses to cannabinoid-based therapies [3,4,5]. However, receptor expression or enzyme abundance should be regarded as candidate determinants rather than established predictive biomarkers, as their association with cannabinoid sensitivity has not yet been consistently validated across independent tumor models.

### 3.3. Oncogenic Dependencies

Tumors may also differ according to their dependence on cannabinoid-modulated survival pathways. PI3K/AKT/mTOR, MAPK, EGFR, and related networks may influence the consequences of cannabinoid-induced signaling perturbation [2,3]. A critical question is whether specific oncogenic dependencies confer enhanced cannabinoid sensitivity.

### 3.4. Metabolic and Stress Vulnerability

Cannabinoids can affect mitochondrial function, oxidative balance, endoplasmic reticulum (ER) stress, autophagy, and cellular metabolism [2,3,14]. Recent primary studies provide mechanistic examples of these effects. In acute myeloid leukemia models, the synthetic cannabinoid derivative WIN-55 induced PARP-dependent cell death accompanied by NAD+ depletion and alterations in glycolytic and pentose phosphate pathway activity, with effects observed in cell lines, primary patient-derived cells, and xenograft models [18]. Similarly, cannabidiol has been shown to alter fatty-acid metabolism and induce CB1 receptor-associated endoplasmic-reticulum stress, reactive oxygen species generation, and mitochondrial dysfunction in ovarian cancer cells [19]. These findings support the concept that metabolic and cellular-stress states may influence cannabinoid responsiveness, but they also illustrate the compound- and tumor-context dependence of these effects.

Tumors with a high basal stress burden or limited metabolic adaptability may therefore be particularly vulnerable to additional cannabinoid-induced stress. However, this remains a mechanistic hypothesis rather than a clinically validated biomarker strategy, and reproducibility across independent models and defined cannabinoid interventions remains to be established.

### 3.5. Compensatory Signaling and Adaptive Resistance

#### 3.5.1. Which Signaling Networks Compensate for Cannabinoid-Induced Stress?

Cannabinoid exposure can perturb cellular homeostasis through oxidative stress, mitochondrial dysfunction, endoplasmic-reticulum stress, autophagy, and alterations in intracellular survival signaling [2,3]. Tumor cells may counteract these perturbations through activation of compensatory survival and stress-response pathways, including PI3K/AKT/mTOR and MAPK signaling, antioxidant responses, autophagy, and metabolic adaptation [2,3]. The relative contribution of these mechanisms is likely to depend on the molecular state of the tumor and the specific cannabinoid intervention.

Importantly, a pathway activated in response to cannabinoid-induced stress may represent both a mechanism of reduced sensitivity and a potential therapeutic vulnerability. Identifying such compensatory responses may therefore provide a mechanistic basis for selecting rational combination partners and candidate biomarkers of resistance.

#### 3.5.2. Does Chronic Exposure Lead to Adaptive Resistance?

Whether chronic cannabinoid exposure induces clinically meaningful adaptive resistance remains an important unresolved question. Tumors that initially respond to cannabinoid-induced stress may undergo adaptive changes in signaling, metabolism, receptor activity, antioxidant capacity, or autophagy that could reduce subsequent treatment sensitivity [2,3]. However, systematic evidence demonstrating acquired resistance specifically to cannabinoid-based anticancer therapy remains limited.

This distinction is important because intrinsic resistance reflects the molecular state of the tumor before treatment, whereas adaptive resistance may emerge under sustained therapeutic pressure. Longitudinal studies incorporating serial molecular profiling could identify treatment-induced changes associated with reduced cannabinoid responsiveness and determine whether emerging resistant states can be therapeutically targeted.

#### 3.5.3. Can Pathway Inhibition Restore or Enhance Cannabinoid Sensitivity?

- Mechanistic rationale:

If compensatory signaling contributes to reduced cannabinoid responsiveness, inhibition of the relevant survival or stress-response pathways may provide a strategy to restore or enhance cannabinoid sensitivity. Cannabinoid-induced perturbation of mitochondrial function, redox homeostasis, endoplasmic-reticulum stress, autophagy, and survival signaling may impose selective pressure on tumor cells to activate compensatory pathways that maintain cellular viability [2,3,14]. These adaptive responses could represent mechanisms of resistance when they effectively buffer cannabinoid-induced stress, but may also create therapeutic vulnerabilities when the tumor becomes dependent on the compensatory pathway [14,15].

- Molecularly guided combination selection:

Rational combination strategies should be guided by the specific biological perturbation induced by the defined cannabinoid intervention and the compensatory response observed in the tumor. Tumors exhibiting increased survival signaling through pathways such as PI3K/AKT/mTOR or MAPK after cannabinoid exposure could be investigated for combination with inhibitors targeting the activated pathway, whereas tumors characterized by enhanced antioxidant capacity, autophagic adaptation, or metabolic compensation may require different combination approaches [2,3,14]. The magnitude and direction of these interactions are likely to depend on the cannabinoid class and molecular target, pharmacological mechanism, dose, treatment sequence, and molecular context [15,16].

- Functional validation:

Functional drug-response studies should determine whether pathway inhibition selectively enhances cannabinoid activity in molecularly defined tumor subsets. Such studies should establish a reproducible interaction between the defined cannabinoid intervention and the combination partner before the strategy is advanced toward clinical testing [7,8,9,20].

- Clinical context:

Clinical evidence for cannabinoid-based combination therapy remains limited and is largely derived from early-phase studies outside urothelial carcinoma. A randomized phase Ib study evaluated nabiximols in combination with temozolomide in patients with recurrent glioblastoma, providing clinical feasibility and preliminary efficacy data but not definitive evidence of anticancer efficacy [13]. The study reported acceptable safety and tolerability, with no apparent effect of nabiximols on temozolomide pharmacokinetics [13]. However, its small sample size and early-phase design limited definitive conclusions regarding therapeutic efficacy, and the study was not designed to identify a molecularly defined subgroup or establish a biomarker-based mechanism of benefit [13].

The subsequent ARISTOCRAT phase II study (NCT05629702) was designed to further evaluate nabiximols plus temozolomide versus placebo plus temozolomide in patients with recurrent MGMT-methylated glioblastoma, illustrating the transition toward more controlled clinical evaluation [21]. However, these studies should not be interpreted as evidence that cannabinoid combinations are broadly effective across tumor types or pharmacological classes [10,15,16,17].

- Requirements for clinical translation:

Combination strategies should therefore be evaluated using a predefined biological hypothesis, a defined cannabinoid intervention, an appropriate combination partner, and biomarkers that identify the relevant molecular vulnerability. Prospective studies should also address dose, treatment sequence, pharmacokinetic/pharmacodynamic interactions, and whether the combination provides incremental clinical benefit over the established standard of care [10,15,17].

The conceptual sequence can therefore be expressed as:

**Defined cannabinoid intervention → molecular perturbation → compensatory pathway activation → actionable vulnerability → rational combination → biomarker-driven clinical validation**

Ultimately, prospective clinical studies will be required to establish whether such biomarker-guided combination strategies improve clinically meaningful outcomes.

## 4. Precision Cannabinoid Oncology: From Molecular Characterization to Biomarker-Guided Clinical Translation

**Figure 1:**
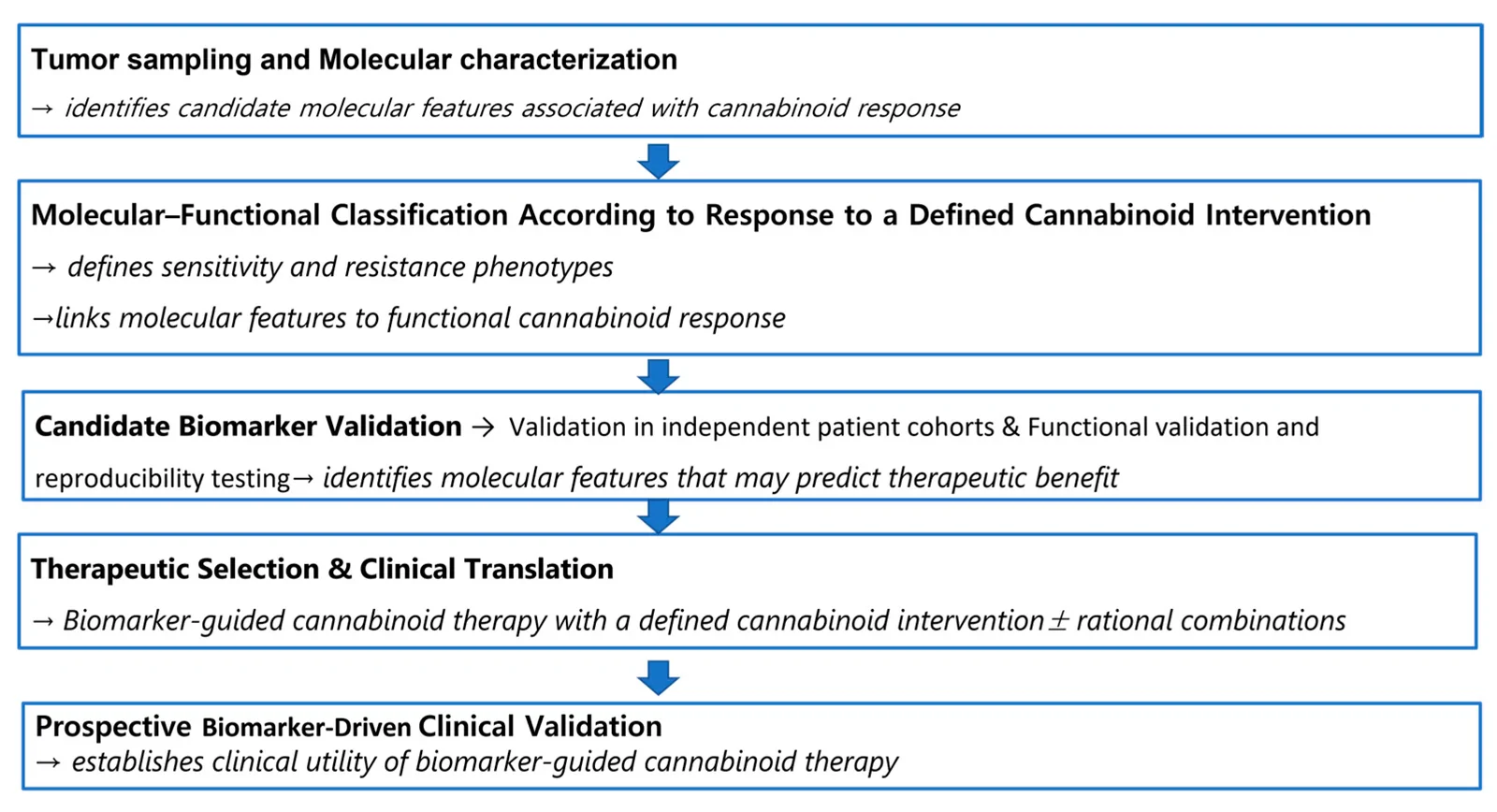
Conceptual framework for precision cannabinoid oncology. A proposed framework for translating the biological activity of defined cannabinoid interventions toward molecularly informed cancer therapy. Tumor sampling and molecular characterization identify candidate molecular features associated with sensitivity or resistance to a specific cannabinoid intervention. Functional response assessment links these molecular features to experimentally observed drug sensitivity, enabling integrated molecular–response classification and candidate biomarker development. Biomarker-guided therapeutic selection may inform evaluation of a defined cannabinoid intervention alone or in rational combinations. Prospective biomarker-driven clinical trials are ultimately required to determine whether these molecular profiles predict differential and clinically meaningful therapeutic benefit and demonstrate clinical utility. Importantly, preclinical biological activity and candidate biomarkers should not be interpreted as evidence of clinical efficacy without prospective clinical validation.

The heterogeneity described above provides the rationale for a precision cannabinoid oncology framework. We propose a sequential strategy progressing through the following steps (Figure 1).

- Tumor molecular characterization: Tumor samples can be characterized using genomic, transcriptomic, proteomic, and metabolomic approaches to identify candidate molecular determinants of cannabinoid response [7]. Key domains may include cannabinoid receptors and endocannabinoid-system components, downstream signaling pathways, metabolic states, cellular stress responses, and features of the tumor microenvironment [2,3,5,11].
- Functional cannabinoid-response assessment: Molecular profiles should be linked to experimentally measured cannabinoid sensitivity using functional assays, organoid models, or other patient-derived systems when available [7,8]. This step provides a functional context for interpreting molecular features and distinguishing biologically sensitive from resistant tumor phenotypes.
- Integrated molecular–response classification: Molecular and functional data should be integrated hierarchically rather than interpreted as independent biomarkers. Linking molecular features to experimentally observed cannabinoid responses may allow tumors to be classified into response-associated biological states.
- Candidate biomarker development and validation: Candidate molecular features can then be evaluated according to their ability to distinguish functionally sensitive from resistant tumors. Importantly, candidate molecular determinants should not be equated with predictive biomarkers. Functional validation and prospective biomarker-driven clinical studies are required to establish whether a molecular feature can identify a patient population with a differential and clinically meaningful therapeutic benefit. Crucially, biological activity observed in preclinical cell culture and animal models must be clearly distinguished from clinically demonstrated therapeutic efficacy, as the former does not guarantee the latter.
- Therapeutic selection and clinical translation: Once response-associated molecular states are established, these profiles could guide therapeutic selection. Patients with a favorable molecular–functional profile could be evaluated in biomarker-driven clinical studies of cannabinoid-based therapy, whereas tumors characterized by compensatory survival pathways could be considered for rational combination strategies.
- Prospective clinical validation: The final step is prospective validation using molecularly characterized patient populations and predefined biomarkers. At present, evidence remains insufficient to establish cannabinoids as routine cancer-directed therapy, underscoring the need for biomarker-driven clinical trials [10].

However, translating preclinical cannabinoid findings into clinical oncology remains challenging. A major translational gap exists between the cannabinoid concentrations often required to induce biological effects in vitro and clinically achievable, non-toxic exposures in humans. In addition, complex in vivo pharmacokinetics, including formulation-dependent bioavailability, tissue distribution, metabolism, and active metabolite formation, may limit the direct translation of preclinical exposure–response relationships to clinical settings. Furthermore, although patient-derived organoids and xenograft models provide valuable functional insights, they may not fully recapitulate the human immune system and tumor microenvironment and therefore may have limited ability to predict clinical response. Accordingly, preclinical biological activity and candidate biomarkers should not be interpreted as evidence of clinical efficacy without prospective clinical validation. These translational considerations support the stepwise framework proposed here, linking preclinical biological activity to molecular determinants and candidate biomarkers, functional validation, clinically relevant exposure and pharmacology, and ultimately prospective biomarker-driven clinical trials.

Thus, the proposed framework aims to shift cannabinoid oncology from the question **“Do cannabinoids work in cancer?”** toward **“Which molecularly defined tumors are most likely to benefit from a specific cannabinoid-based intervention?”**

## 5. Challenges and Future Directions

Several barriers must be overcome before precision cannabinoid oncology can be implemented clinically.

### 5.1. Pharmacological Standardization

Cannabinoid interventions differ substantially in compound composition, receptor activity, formulation, dose, route of administration, bioavailability, and pharmacokinetic properties [1,3,10]. Therefore, phytocannabinoids, synthetic cannabinoid analogs or receptor agonists, and endocannabinoid-modulating agents should not be assumed to share the same molecular determinants of therapeutic response. Biomarker studies must consequently be linked to a clearly defined cannabinoid intervention. Differences in formulation, route of administration, dose, and bioavailability may influence systemic exposure and pharmacokinetic profiles [1,3,10]. Importantly, the therapeutic relevance of preclinical findings depends not only on pharmacodynamic activity but also on whether effective concentrations can be achieved within an acceptable safety and therapeutic window. Potential adverse effects, drug–drug interactions, and pharmacokinetic/pharmacodynamic differences may further influence the feasibility of cannabinoid-based treatment, particularly in combination strategies. Moreover, concentrations producing biological effects in preclinical models may not necessarily correspond to clinically achievable exposure [1,3,10]. Standardized formulations and harmonized pharmacokinetic/pharmacodynamic assessments will therefore be important for determining whether molecular–response associations are reproducible under defined treatment conditions and can be translated into clinically relevant therapeutic strategies [10].

### 5.2. Biomarker Validation and Resistance

- Functional validation: The major challenge is distinguishing molecular features that merely correlate with cannabinoid exposure from those that actually determine therapeutic vulnerability. Functional validation should therefore accompany multi-omic profiling [7,8]. Biomarker development should follow a hierarchical process in which target expression or pathway activity is first linked to a reproducible functional phenotype and subsequently evaluated for its ability to predict therapeutic benefit in clinically relevant models [7,8].
- Candidate versus predictive biomarkers: A molecular feature should initially be considered a candidate biomarker when it is associated with cannabinoid sensitivity or resistance but has not yet demonstrated predictive clinical utility. Advancement to a predictive biomarker requires reproducible association with response, mechanistic or functional validation, and evidence that the biomarker identifies a subgroup with a differential probability of benefit from a defined cannabinoid intervention [22]. Reproducibility should be assessed across independent experimental systems, defined cannabinoid interventions, and clinically relevant exposure ranges before clinical evaluation.
- Composite biomarker profiles: A composite molecular profile may ultimately prove more informative than any single marker, particularly given the context-dependent and pleiotropic effects of cannabinoid signaling [2,3]. Such profiles may integrate receptor and endocannabinoid-system activity with downstream signaling, metabolic state, cellular stress, and tumor–microenvironmental features.
- Longitudinal assessment and resistance: Longitudinal sampling will be important for distinguishing intrinsic resistance from treatment-induced adaptation [23]. Serial molecular profiling could reveal changes in signaling, metabolism, receptor activity, or the tumor microenvironment that emerge during therapy [23]. Such longitudinal analyses may help identify acquired resistance mechanisms and refine biomarker-defined treatment strategies over the course of therapy.
- Prospective clinical validation: Ultimately, candidate biomarkers must be prospectively evaluated to determine whether they predict differential therapeutic benefit from a predefined cannabinoid intervention. Appropriate studies should incorporate prespecified biomarker thresholds and treatment-by-biomarker interaction analyses against an appropriate comparator [22].

### 5.3. Rational Combination Strategies

The multi-target nature of cannabinoids provides a rationale for combination therapy, particularly when cannabinoid-induced stress activates compensatory survival pathways. Preclinical evidence supports investigation of combinations with chemotherapy and other anticancer therapies, although effects vary according to tumor and treatment context [1,2,3].

Early clinical studies have also explored cannabinoid combinations, including nabiximols plus temozolomide in recurrent glioblastoma [13,21]. These studies illustrate the progression toward more rigorous clinical evaluation but do not establish broad clinical efficacy. Future combinations should therefore be selected according to molecular vulnerability and compensatory pathway activation, rather than empirical drug pairing.

### 5.4. Prospective Biomarker-Driven Trials

Ultimately, clinical translation requires prospective validation. Current clinical evidence for cannabinoids as cancer-directed therapy remains limited, and the 2024 ASCO guideline concluded that evidence was insufficient to recommend cannabis or cannabinoids as cancer-directed treatment outside clinical trials [10]. Importantly, symptomatic benefits, such as management of refractory chemotherapy-induced nausea and vomiting, should not be conflated with direct antitumor activity or survival benefit [10].

Future trials should therefore incorporate predefined molecular biomarkers, standardized cannabinoid interventions, pharmacokinetic/pharmacodynamic assessment, and molecularly characterized patient populations. The goal should be to determine whether biomarker-selected patients derive clinically meaningful benefit from a defined cannabinoid-based therapy.

## 6. Conclusions

Cannabinoids represent a potential therapeutic opportunity in oncology because their biological effects extend across multiple signaling, metabolic, stress-response, and tumor–microenvironmental pathways. This multi-target activity, however, also creates substantial heterogeneity in therapeutic response and makes a universal biomarker unlikely.

The central challenge for cannabinoid oncology is therefore to identify which molecularly defined tumors are vulnerable to specific cannabinoid-based interventions. Integrated molecular profiling, functional response assessment, and prospective biomarker validation may provide a foundation for identifying such tumor subsets and for developing rational combination strategies.

The proposed framework shifts cannabinoid oncology from empirical investigation toward molecularly informed patient selection, analogous to the broader evolution of precision oncology. Rather than assuming that cannabinoid activity is broadly applicable across malignancies, therapeutic development should focus on defining the biological contexts in which cannabinoid-mediated signaling perturbation produces clinically meaningful benefit.

At present, however, precision cannabinoid oncology remains a prospective framework rather than an established clinical approach. The lack of validated predictive biomarkers, limited clinical evidence of anticancer efficacy, uncertainty regarding clinically relevant exposure, and the need for prospective biomarker-driven trials remain important barriers to clinical translation. Ultimately, the potential of this approach will depend on whether candidate molecular determinants can be validated as reproducible, clinically actionable predictive biomarkers. If prospectively validated, precision cannabinoid oncology could support the development of cannabinoid-based therapies as biomarker-guided treatment options for molecularly defined subsets of patients.

## Figures and Tables

**Table 1:** Cannabinoid-mediated modulation of Hallmarks of Cancer and associated signaling pathways.

Hallmark of Cancer	Major Biological Effects	Representative Pathways
Proliferative signaling	Growth inhibition, cell-cycle arrest	EGFR, PI3K/AKT, RAS/RAF/ERK, mTOR
Inactivation of growth suppressors	Restoration of cell-cycle control	p53, p21, p27
Resistance to cell death	Apoptosis, autophagy induction	Mitochondrial pathways, oxidative stress
Deregulated metabolism	Metabolic reprogramming	Mitochondrial function
Angiogenesis	Reduced vascular development	VEGF-related pathways
Invasion/metastasis	Reduced migration and invasion	EMT-associated pathways
Immune evasion/TME	Immune modulation	Cytokines, immune signaling, microbiome

Abbreviations: AKT, protein kinase B; EMT, epithelial–mesenchymal transition; EGFR, epidermal growth factor receptor; ERK, extracellular signal-regulated kinase; mTOR, mechanistic target of rapamycin; PI3K, phosphoinositide 3-kinase; RAS, rat sarcoma protein; RAF, rapidly accelerated fibrosarcoma; TME, tumor microenvironment; VEGF, vascular endothelial growth factor.

**Table 2:** Representative cancer types, cannabinoid interventions, proposed mechanisms, candidate biomarkers, and evidence level.

Cancer Type	Cannabinoid Intervention	Proposed Mechanism	Candidate Molecular Features	Evidence Level
Glioblastoma	Nabiximols (THC:CBD)	Cannabinoid receptor signaling; ER stress; interaction with temozolomide [13,14,15]	CNR1/CNR2 expression/activity; MGMT status; stress-response pathways	Preclinical + early clinical
Breast cancer	THC/CBD and synthetic cannabinoids	CB receptor signaling; apoptosis; autophagy; oxidative stress [2,14,16]	CNR1/CNR2, FAAH/MGLL expression/activity; PI3K/AKT and/MAPK; metabolic/stress signatures	Predominantly preclinical
Prostate cancer	Cannabinoids; endocannabinoid system modulation	Endocannabinoid metabolism; lipid signaling; survival pathways [5]	FAAH/MGLL expression/activity; CNR1/CNR2; lipid-metabolic state	Predominantly preclinical
Pancreatic cancer	Cannabinoid receptor agonism/cannabinoids	ER stress; autophagy; survival signaling [14]	CNR1/CNR2; ER-stress/autophagy signatures; metabolic state	Predominantly preclinical
Other solid tumors	Various cannabinoids	Context-dependent receptor signaling and stress responses [2,3,14,17]	ECS activity; signaling/metabolic/stress signatures	Predominantly preclinical

Abbreviations: THC, Δ9-tetrahydrocannabinol; CBD, cannabidiol; ER, endoplasmic reticulum; MGMT, O6-methylguanine-DNA methyltransferase; ECS, endocannabinoid system. Note: Evidence levels distinguish predominantly preclinical evidence from settings with limited early clinical evaluation and should not be interpreted as evidence of definitive clinical efficacy. Candidate molecular features are hypothesis-generating and should not be interpreted as clinically validated predictive biomarkers. Early clinical evidence indicates clinical evaluation but does not establish therapeutic efficacy or biomarker-defined benefit.

## Data Availability

No new data were created or analyzed in this study. Data sharing is not applicable to this article.
